# The Pathobiology of IL-11 in Kidney Disease: From Epithelial Cell to Fibroblast and Back Again

**DOI:** 10.1016/j.ajpath.2023.09.011

**Published:** 2023-10-09

**Authors:** Anissa A. Widjaja, Stuart A. Cook

**Affiliations:** ∗ Cardiovascular and Metabolic Disorders Program, Duke-National University of Singapore Medical School, Singapore; † National Heart Research Institute Singapore, National Heart Centre Singapore, Singapore; ‡ MRC-London Institute of Medical Sciences, Hammersmith Hospital Campus, London, United Kingdom

Up to 25% of adults >60 years of age have chronic kidney disease (CKD), which represents a growing global health challenge. Some progress has been made in treating kidney diseases in recent years, but CKD for the most part remains progressive and leads to end-stage kidney disease, with the subsequent need for renal replacement therapy (life-long dialysis or renal transplantation). Although CKD can arise from a multitude of factors, including infections, obstructions, toxins, genetics, hemodynamics, and metabolic issues, renal fibrosis emerges as a common consequence of each of these triggers. Of all the CKD pathologies, extent of fibrosis most accurately predicts progression to end-stage kidney disease and poor clinical outcomes.

## Epithelial Stromal Crosstalk in Renal Fibrosis

The renal parenchyma exhibits remarkable adaptability, which reflects its complex embryonic origins, and it can undergo profound repair and regeneration after kidney injury, which is driven by proliferation of renal tubular epithelial cells (RTECs). However, after repeated or sustained injury, regeneration of RTECs fails, and this primary pathology leads to subsequent renal fibrosis and kidney failure.^1^

Acting at the interface of regeneration and fibrosis is epithelial mesenchymal transition (EMT) of RTECs, a process that is strongly controlled by an E-cadherin repressor, SNAI1.^2^ Once SNAI1 is up-regulated in damaged RTECs, they are redirected to a dedifferentiated mesenchymal state, lose their specialized functions, and secrete a range of profibrotic factors that act in paracrine on underlying renal fibroblasts. SNAI1 itself undergoes posttranscriptional regulation mediated by glycogen synthase kinase-3β, which phosphorylates SNAI1 at two sites, promoting its ubiquitination, exclusion from the nucleus, and degradation.^3^ Control of the E-cadherin:SNAI1 switch is provided by extracellular signal–regulated kinase (ERK) and p90RSK, which phosphorylate glycogen synthase kinase-3β at Thr43 and Ser9, respectively, resulting in glycogen synthase kinase-3β inactivation.^4^ Of note, SNAI1 up-regulation in the liver is associated with failed hepatocyte regeneration and liver fibrosis, suggesting a more widespread role in organ fibrosis and failure.^5^

There was confusion and controversy about the extent to which RTECs undergo EMT in the earlier literature as these cells do not migrate to become myofibroblasts. It has therefore been suggested that RTECs undergo partial EMT (pEMT) or enter a “failed-repair proximal tubule cell” state.^6^ In light of this, therapeutic targeting of pEMT is proposed as an approach to promote kidney repair and regeneration as it targets a primary pathology underlying renal failure rather than addressing renal fibrosis, which is consequential. Clinical trials of anti–transforming growth factor β (TGF-β), a potent driver of renal fibrosis and pEMT, were initiated in patients with CKD.^7^ Unfortunately, these trials failed due to their lack of effectiveness,^7^ perhaps related to dose-limiting toxicities,^8^ highlighting the need for new therapeutic targets to treat CKD.

## IL-11 EMT and Kidney Disease

IL-11 is a member of the IL-6 family of cytokines. Across varied species, IL-11 is up-regulated in the kidney in response to insults such as high blood pressure,^9^ diabetes,^10^^,^^11^ reduced blood flow,^12^ exposure to toxins,^13^ infection,^14^ and urinary tract obstruction.^12^ In humans, elevated urinary IL-11 levels have been observed in patients with nephropathy and nephritis, and IL-11 ranks among the most up-regulated genes in tissue slices isolated from individuals with end-stage kidney disease.^15^^,^^16^

In 2017, our group was the first to describe a profibrotic effect of IL-11 in the kidney. This finding challenged the earlier literature, which suggested the opposite to be true.^13^ At that time, data highlighted that IL-11–induced renal fibrosis and dysfunction were ERK related and mediated primarily via its activity in renal fibroblasts, and no connection to RTECs or pEMT was made.

We previously examined the role of IL-11 in kidney disease in a mouse model of Alport syndrome, which is a disease of the renal glomerulus caused by mutation in collagen type IV.^17^ IL-11:EGFP reporter mice were crossed to Alport syndrome mice (129-Col4a3tm1Dec/J or *Col4a3*^*−/−*^), which resulted in specific up-regulation of IL-11 in RTECs that was accompanied by increased SNAI1 expression. This up-regulation indicates pEMT state, as well as renal fibrosis, kidney failure, and early death. These pathologies were collectively mitigated by anti–IL-11 therapy, thus further confirming the mechanistic importance of IL-11 in renal disease. This study identified IL-11 as a therapeutic target and established a first link between IL-11 and RTEC pEMT in the diseased kidney.

We also examined the effects of IL-11 in a mouse model of CKD with specific focus on renal repair and regeneration.^18^ IL-11 drove ERK/P90RSK–dependent SNAI1 up-regulation in RTECs, resulting in withdrawal of RTECs from the cell cycle and their transition into a dysfunctional pEMT state. TGF-β–stimulated RTEC pEMT was also shown to be IL-11 dependent and reversible using neutralizing IL-11 antibodies. Remarkably, anti–IL-11 administered to mice with CKD reversed pEMT phenotypes and stimulated stalled RTECs to re-enter the cell cycle and proliferate, which resulted in renal repair and regeneration.

Taken as a whole, it is apparent that the earlier literature pointed to a possible role of IL-11 in renal disease, a notion that has now been established through a range of *in vitro* and *in vivo* models. Very recently, our understanding of the pathobiology of IL-11 in the kidney has expanded beyond the fibroblast and fibrosis to encompass its role in the epithelium and pEMT, which act together in a vicious cycle of self-amplifying tubular and parenchymal dysfunction, fibrosis, and failure. This likely explains the large effect of IL-11 inhibition in kidney disease as it addresses at least two cellular components of disease pathology: the stroma and the epithelium.

## Contribution of the Current Published Study

In the study published in the current issue, Li et al^19^ examined the impact of IL-11 on RTEC pEMT and determined the effects of micheliolide (MCL), a more stable derivative of parthenolide. MCL has recently been shown to have anti-inflammatory properties, on IL-11–related pathologies in models of renal fibrosis and failure.

In a mouse model of unilateral ureteric obstruction, the authors observed an up-regulation of *Il11* and *Il11ra1* RNA that was mostly localized to RTECs.^19^ Administration of *Il11* shRNA reduced pathogenic signaling and fibrosis while restoring E-cadherin levels, whereas overexpression of exogenous mouse IL-11 exacerbated unilateral ureteric obstruction–induced pathologies. *In vitro*, TGF-β stimulated RTEC to enter a pEMT state that was reduced by Il11 shRNA. The authors proceeded to show that IL-11 stimulation causes RTECs to enter G2/M cell cycle arrest while down-regulating RTEC-specific genes and up-regulating EMT genes (*Snai1*, *Twist*, and *Mtdh*).

Using virtual molecular docking, the authors discovered that MCL could form hydrogen bonds at key sites on IL-11 necessary for IL-11 receptor subunit alpha (IL-11RA) binding.^19^ In pull-down experiments, it was shown that biotin-labeled MCL interacted with IL-11 and that MCL treatment interfered with IL-11:IL-11RA binding. In RTECs, MCL (5 μmol/L) inhibited IL-11–stimulated STAT3 and ERK1/2 phosphorylation, MTDH, fibronectin, and collagen type I up-regulation, and restored E-cadherin levels. *In vivo*, administration of dimethylaminomicheliolide, a pro-drug of MCL, reduced unilateral ureteric obstruction–induced renal fibrosis that was associated with lesser pEMT and a reduction in IL-11–related pathologies.

Overall, the study by Li et al^19^ provides robust replication and strong confirmation of the earlier studies^17^, ^18^ showing IL-11 to be a major determinant of RTEC pEMT and that IL-11 causes impaired renal repair, renal fibrosis, and renal failure. The use of shRNA loss-of-function and plasmid-based gain-of-function approaches *in vivo* provide orthogonal support for IL-11 effects. This complements the existing body of data, which includes genetic gain-of-function and loss-of-function approaches, as well as experiments using anti–IL-11 or anti–IL-11RA therapy.^17^^,^^18^

The authors conclude that MCL represents an inhibitor of IL-11 signaling and that this mechanism could underlie the antifibrotic effects of MCL.^19^ Although this may be the case, MCL is known to inhibit NF-κB and has strong anti-inflammatory effects directly in immune cells, including on the phosphatidylinositol 3-kinase/AKT axis. IL-11RA is lowly expressed on immune cells^13^ and has inconsistent effects on the phosphatidylinositol 3-kinase/AKT axis.^20^ Hence, it is plausible that the effects of MCL, at least in part, are unrelated to inhibition of IL-11 signaling. IL-11 is a very proline-rich protein, which may cause nonspecific binding of MCL to IL-11 and confound the pull-down studies.

In summary, the study by Li et al^19^ in the current issue of *The American Journal of Pathology* provides a compelling validation and extension of recent findings: IL-11 is a renal disease factor that has major adverse effects on RTECs by causing them to enter a nonproliferative, dedifferentiated, SNAI1-expressing pEMT state that leads to renal fibrosis and failure.^13^^,^^17^^,^^18^

**Figure 1:**
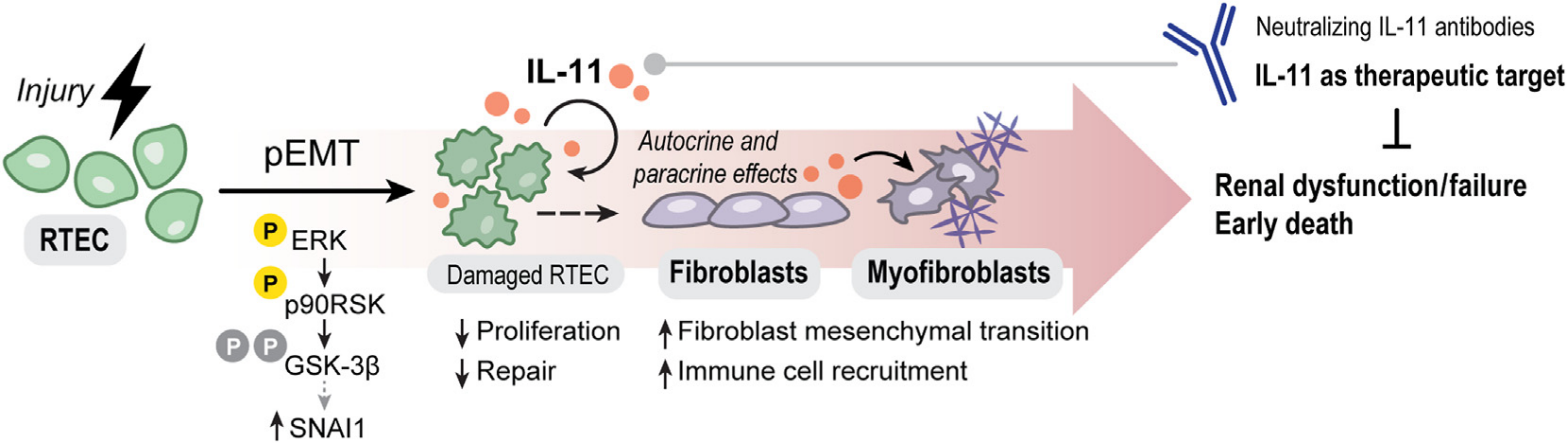
Proposed mechanisms by which IL-11 induces renal dysfunction in kidney disease. In response to injury, damaged renal tubular epithelial cells (RTECs) up-regulate IL-11, which activates the extracellular signal–regulated kinase (ERK)/p90RSK/glycogen synthase kinase-3β (GSK-3β) pathway. This leads to an autocrine SNAI1-mediated program of partial epithelial-to-mesenchymal transition (pEMT) of RTECs. IL-11 secreted from RTECs also acts in paracrine manner to recruit immune cells and drive fibroblast-to-myofibroblast transformation, resulting in kidney fibrosis. Neutralizing antibodies against IL-11 reduce renal dysfunction and extend life expectancy in some mouse models, thus proposing IL-11 as a therapeutic target for treating chronic kidney disease.

IL-11 also has major profibrotic activity directly in fibroblasts, which most highly express IL-11RA. Hence, in the diseased kidney, IL-11 released from damaged RTECs can act in autocrine fashion to further inactivate RTECs and in paracrine manner to stimulate renal fibroblast-to-myofibroblast transformation, which amplifies renal IL-11 levels and disease pathobiology (Figure 1). IL-11 also stimulates an ERK/P90RSK/glycogen synthase kinase-3β axis of SNAI1-related mesenchymal transition of RTECs, fibroblasts, and other cells^21^ (Figure 1), suggesting this as a primary and conserved function of IL-11, possibly related to its evolutionary ancient role in epimorphic appendage regeneration in fish.^22^

Although IL-6 and TGF-β are two of the most studied genes in biomedical science, IL-11, by comparison, is vastly understudied.^23^^,^^24^ The earlier literature suggested IL-11 to be cytoprotective, but recent evidence seems to indicate the opposite.^25^ In addition, with studies now replicating IL-11 as a therapeutic target for lung,^26^ liver,^27^ and kidney^19^ fibrosis, the stage seems set for greater activity in the field of IL-11 research. In the kidney, there remains an outstanding question pertaining to the effects of IL-11 on inflammatory cells: although inhibition of IL-11 signaling clearly reduces kidney inflammation in renal disease, it is not known whether this effect is a direct or indirect consequence of IL-11 action. In addition, the role of IL-11 and IL-11RA in podocytes is equally important and yet remains unexplored.

Although anti–TGF-β is highly efficient in inhibiting the CKD progression in mice, clinical trials targeting TGF-β (LY2382770 and fresolimumab) were unsuccessful. Inhibition of IL-11, either by antibody therapy or small molecules, may provide a novel and potentially safe approach for patients with CKD as it addresses disease pathology across multiple cellular compartments.^22^ Currently, there are three registered phase 1 clinical trials of anti–IL-11/IL-11RA therapies (ClinicalTrials.gov, https://clinicaltrials.gov, last accessed September 20, 2023) (Table 1); although these trials are focused on safety and fibrotic lung disease, the availability of this new therapeutic class may pave the way for clinical trials targeting kidney disease in the future.

**Table 1:** Anti–IL-11 or anti–IL-11RA Clinical Trials Registered at ClinicalTrials.gov as of September 20, 2023

Clinical trial ID	Sponsor	Last updated	Target enrollment
NCT05658107	Boehringer Ingelheim	July 28, 2023	64
NCT05331300	Lassen Therapeutics	July 27, 2023	80
NCT05740475	Mabwell Bioscience	June 13, 2023	32

All data released to the public via this interface. IL-11RA, IL-11 receptor subunit alpha.

## Disclosure Statement

A.A.W. and S.A.C. are co-inventors of the patents US11339216B2 and WO2021255182A1, which have been licensed to Boehringer Ingelheim and VVB Bio, respectively. S.A.C. is inventor on the patents WO/2018/109174 and WO/2018/109170. S.A.C. is a co-founder and shareholder of Enleofen Bio Pte Ltd and VVB Bio Pte Ltd.
